# Lithium-Associated Hypercalcemia Presenting with Neuropsychiatric Manifestations in a Patient with Bipolar Disorder

**DOI:** 10.1155/2020/6630838

**Published:** 2020-12-10

**Authors:** Roukaya Benjelloun, Imane Motaib, Yassine Otheman

**Affiliations:** ^1^Faculty of Medicine, Mohammed VI University of Health Sciences (UM6SS), Casablanca, Morocco; ^2^Faculty of Medicine and Pharmacy, Sidi Mohamed Ben Abdellah University, Fez, Morocco

## Abstract

One of the overlooked adverse effects of lithium treatment is neuropsychiatric manifestations induced by hypercalcemia (LAH). Here, we present the case of a patient, with bipolar disorder under lithium, who presented with neuropsychiatric symptoms that revealed hyperparathyroidism-induced hypercalcemia. This case illustrates the importance of monitoring parathyroid function and calcium levels in patients under lithium, especially in premenopaused women. LAH may mimic symptoms of manic episodes and should be sought in the case of brutal onset of confusion, anxiety, and/or hallucinations in the course of a bipolar disorder.

## 1. Introduction

Lithium is a gold standard mood stabilizer, known for its potential toxicity and severe side effects. One of the overlooked, but not so rare, adverse effects is lithium-associated hypercalcemia (LAH) that can mimic psychiatric conditions and be mistaken for bipolar disorder relapse [1]. The mechanism by which lithium induces hypercalcemia is not fully elucidated. The common thread of all hypotheses is the elevation of parathormone level, which is the main regulator of calcium homeostasis. It is very likely that lithium inhibits the calcium-negative feedback on the parathormone secretion [2]. More precisely, lithium tends to antagonize the calcium sensing receptor (CASR) and, in doing so, raises the threshold and increases the set point of the required calcium level to inhibit parathormone secretion [3]. Here, we present the case of a female patient with bipolar disorder (BD) under lithium, who presented with neuropsychiatric symptoms that revealed hyperparathyroidism-induced hypercalcemia.

## 2. Case Report

A 68-year-old female patient with a history of BD, maintained under lithium carbonate for 24 years, presented to the emergency room with brutal onset of subconfusion, insomnia, and visual hallucinations. According to her relatives, the patient was diagnosed with BD after a manic episode for which she was admitted in a psychiatric unit when she was in her late twenties. Afterward, she presented mainly manic and hypomanic episodes. Five years earlier, the patient's referring psychiatrist put the patient under 500 mg lithium carbonate monotherapy, and she had been stable all this period.

At admission, the patient neuropsychiatric evaluation found vivid visual hallucinations with onirism and confusion. Electrocardiogram and brain MRI were normal. Calcitonin, thyroid stimulating hormone (TSH), ionogram, azotaemia, and creatinine were normal. The patient's total serum calcium level after correction was high (127 mg/l [normal: 88–105]) and the serum parathormone level (PTH) was five times normal values (380 pg/ml [normal: 8.7-79.6]). Urine methoxylated derivatives were negative, which eliminated pheochromocytoma. A cervical scan suspected a right lower parathyroid tumor, confirmed by cervical ultrasonography that found a 12 × 8 mm adenoma.

The patient received furosemide (120 mg per 24 hours), zoledronic acid (4 mg in a single dose), and parenteral rehydration. Lithium therapy was discontinued. Five days after her admission, and even if the serum calcium level decreased to 93 mg/l, psychiatric symptoms got worse: the patient showed anxiety, subagitation, visual hallucinations, and incoherent speech. The patient underwent urgent parathyroidectomy, and postoperative evolution was favorable. PTH levels were monitored on days 2, 5, 7, and 15 and then a month later. Anatomopathological study confirmed the radiological diagnosis of parathyroid adenoma.

## 3. Discussion

In 1973, Garfinkel et al. reported the first description of lithium-associated hypercalcemia (LAH) [4]. Even if LAH is described as rare, a meta-analysis yielded by McKnight et al. showed that patients under lithium therapy had an absolute risk of 10% to develop primary hyperparathyroidism attributable to lithium [5]. In patients under lithium with a history of BD, LAH may present with neuropsychiatric symptoms, such as anxiety, depression, psychosis, and hallucinations [6] and, therefore, be mistaken for a bipolar relapse. The main explanation to hypercalcemia-induced psychiatric symptoms is that patients tend to show an elevation of calcium levels in the cerebrospinal fluid, which is suspected to alter central monoamine metabolism [7].

After we discontinued lithium therapy in this patient, the serum calcium level started to decrease. However, we reached full clinical and biological stabilization only after the right lower parathyroid was removed. If lithium therapy may induce mild and reversible hyperparathyroidism that can regress after lithium cessation [8], in most cases, discontinuing lithium has no impact on calcium homeostasis, and surgery is considered as the best solution [9].

Parathyroid function monitoring, in patients under lithium, has been included in the NICE (National institute of Health and Care excellence, UK) guidelines since 2006 [10]. Moreover, the International Society for Bipolar Disorder (ISBD) recommended controlling calcium levels at the initiation of lithium treatment, after 6 months, and once a year thereafter [11].

This case illustrates the importance of monitoring parathyroid function and calcium levels in all patients under lithium. Moreover, LAH may mimic symptoms of BD relapse and should be sought in cases of brutal onset of confusion, anxiety, and/or hallucinations.
